# Light Wines and Temperance Drinks

**Published:** 1907-06-01

**Authors:** 


### LIGHT WINES AND TEMPERANCE DRINKS.

### Their Chemical Composition and Physiological Action.

At the present time, when the question of alcoholic beverages is allotted so much space in both lay and medical papers, one feature of the controversy must strike every careful reader. This feature is that while in the copious articles recently published upon this subject theories and opinions abound, facts are lamentably absent. In the language of a distinguished living chemist, "it is far easier to scratch one's head than to burn one's finger," or in other words to discuss philosophically what may be than to go into the laboratory and find out what is. In the report which we intend to publish shortly we have first of all confined our attention to the action of alcohol, and have shown clearly that this substance when taken in quantities corresponding to moderate wine drinking, has a favourable influence upon the digestive processes, helps us to get the most value out of a given quantity of food, and must in every way be regarded as a proper and profitable constituent of the diet calculated to fulfil the exigencies of modern life.

The report deals next with the chemical properties and dietetic effects of the light table wines. For this purpose we have examined an exhaustive series of clarets, moselles, hocks, and champagnes, and have shown by ascertained facts the beneficial influence which these beverages exert upon each stage of the complicated process of digestion. This inquiry has enabled us further both to separate wheat from chaff, or good wine from bad, and to indicate the general principles which should guide the medical profession in directing the use of different wines to different individuals. Last but not least we have been able to point out in general terms the amount of wine which is beneficial and the amount likely to be injurious.

The report concludes with an experimental inquiry into the effects of tea and temperance drinks upon digestion, and shows clearly how favourably wines compare with the above beverages.

THE HOSPITAL *June* 1, 1907.

